# Association of Electrical Cardioversion with Brain Perfusion and Cognitive Function in Patients with Atrial Fibrillation

**DOI:** 10.3390/life13040935

**Published:** 2023-04-03

**Authors:** Josip Kedžo, Sanja Lovrić Kojundžić, Maja Marinović Guić, Leida Tandara, Toni Brešković, Zrinka Jurišić

**Affiliations:** 1 Department of Cardiology, University Hospital of Split, 21000 Split, Croatia; jkedzo@kbsplit.hr (J.K.);; 2 Department of Diagnostic and Interventional Radiology, University Hospital of Split, 21000 Split, Croatia; 3 University Department of Health Studies, University of Split, 21000 Split, Croatia; 4 School of Medicine, University of Split, 21000 Split, Croatia; 5 Medical Laboratory Diagnostic Division, Medical Biochemistry and Laboratory Medicine Subdivision, University Hospital of Split, 21000 Split, Croatia

**Keywords:** magnetic resonance, cognition, atrial fibrillation, brain perfusion

## Abstract

Background: The association of atrial fibrillation (AF) and brain perfusion (BP) has not been well-defined. This study aimed to determine the association of persistent AF with BP and cognition, in comparison to control subjects and with regards to electrical cardioversion (ECV). Methods: This study compared 25 patients with persistent AF undergoing elective ECV with 16 age/sex-matched controls. We measured regional BP by using the magnetic resonance (MRI) arterial spin labelling technique. Cognitive function was assessed using the Patient-Reported Outcomes Measurement Information System (PROMIS) cognitive function index. Measurements were performed at baseline and 6 weeks after ECV. Results: There was no significant difference in BP between AF patients and control subjects (*p* > 0.05). Following the ECV, there was a significant improvement in BP in 15 patients who maintained sinus rhythm, while there was no significant change in the recurrence group (297 ± 24 before vs. 328 ± 37 after ECV, *p* = 0.008, and 297 ± 22 before vs. 307 ± 24 after ECV, *p* = 0.45, respectively). There was no difference in the cognitive assessment between AF patients and control subjects, as well as before and after ECV within the AF group (52.2 ± 9.6 vs. 51.2 ± 6.2, *p* = 0.71 and 53 ± 10 vs. 54 ± 9, *p* = 0.46, respectively). Conclusions: This study did not show difference in BP between persistent AF patients and matched control subjects. Restoration of sinus rhythm was associated with significantly improved BP. There was no association of ECV and changes in cognitive function.

## 1. Introduction

Atrial fibrillation (AF) is the most common clinically significant arrhythmia that is associated with an increased risk of all-cause and cardiovascular mortality [1]. Its incidence is emerging with projections of more than a two-fold increase in the upcoming decades [2]. Ischaemic stroke is one of the most devastating complications of AF [3], leading to frailty, disability, and increased risk of dementia [4]. However, irrespectively of the stroke, AF is associated with cognitive and psychomotor impairment [5]. A recent meta-analysis of prospective population studies estimated that AF carries a 45% risk of cognitive impairment [6]. Proposed underlying mechanisms include a proinflammatory and prothrombotic state, silent cerebral infarcts, cerebral microbleeds, and brain hypoperfusion, all of which can ultimately result in brain atrophy [7]. An additional cross-sectional study reported a smaller brain volume and lower cognitive performance amongst individuals with AF, including the strong dependence on the arrhythmia duration [8]. Consistent results were reported in a cohort of elderly patients with persistent AF that exhibited a reduced brain volume, cerebral blood flow, and estimated brain perfusion measured by phase contrast magnetic resonance imaging (MRI), compared to subjects in sinus rhythm [9].

Furthermore, it was shown that cardioversion to sinus rhythm improves brain perfusion measured by arterial spin labelling (ASL) MRI, while there was no effect following unsuccessful cardioversion [10]. However, there is an unmet need for more literature evidence, particularly regarding the confounding bias of co-existent patient characteristics and comorbidities, different burden of AF across patient groups, and extensity of cerebral perfusion analysis. It remains unclear which AF patients benefit the most from the rhythm control strategy in terms of brain perfusion parameters. Whether the level of AF burden is associated with alterations in brain perfusion is unknown. Finally, it remains unknown to what extent other cardiovascular risk factors additionally contribute to reduced brain perfusion, in addition to AF-related mechanisms, such as heart rate variability and lack of atrial contraction. Common cardiovascular risk factors, such as age, sex, arterial hypertension, cholesterol level, smoking status, diabetes mellitus, and obesity have been independently associated with the level of cerebral perfusion [11].

Therefore, the aim of our study was to determine whether electrical cardioversion (ECV) to sinus rhythm improves brain perfusion in patients with recently diagnosed persistent AF, and whether there are differences in brain perfusion between AF patients and control subjects with similar cardiovascular risk profile without AF.

## 2. Materials and Methods

### 2.1. Ethical Considerations and Informed Consent

The study was conducted according to the *Helsinki Declaration* and approved by the Medical Research Ethical Committee of the University Hospital of Split (No. 2181-147/01/06/M.S.-21-02). All included subjects have provided an informed written consent prior to the study initiation. All the procedures within the study have been conducted in line with the principles of Good Clinical Practice.

### 2.2. Study Design and Patients

The observational prospective study was conducted at the University Hospital of Split between January and November 2021. We enrolled 25 subjects with persistent AF undergoing elective ECV and 16 age- and sex-matched control subjects (Supplementary Figure S1). All enrolled subjects were older than 18 years, and they conformed to the study protocol, including the laboratory analysis, brain MRI, and cognitive assessment. The diagnosis of persistent AF was established according to the guidelines of the European Society of Cardiology [12]. Rhythm control strategy was at the discretion of the referring cardiologist, prior to screening for the study.

Exclusion criteria of the study were: long-standing persistent AF (>1 year duration of ongoing episode); atrial flutter; left ventricular ejection fraction < 40%; severe valvular disease; history of stroke/transient ischemic attack; intervention on carotid arteries; diagnosis of significant (>70%) stenosis of carotid arteries on imaging or presence of carotid bruit on physical examination; cognitive disorders or dementia; any diagnosed neurologic disease; and any diagnosed psychiatric disorder. Additionally, patients with cardiac implantable electronic devices or other contraindications for MRI were not considered for the study enrollment.

The control group consisted of patients without a history of AF or any other arrhythmia. We included patients who had a similar cardiovascular risk profile, such as age, sex, smoking, hypertension, obesity, and diabetes mellitus. They were recruited from the general cardiology outpatient clinic, where they were referred by a family medicine doctor for various reasons. Their electrocardiogram (ECG) was recorded on the day of MRI to confirm sinus rhythm. Control patients underwent only one MRI examination. The exclusion criteria were the same as for the study group.

### 2.3. Outcomes

The primary outcomes of this study were the comparisons of regional brain perfusion measured by arterial spin labelling MRI between patients with persistent AF and control subjects and within patients with persistent AF, before and 6 weeks after the electrical cardioversion. Secondary outcomes included: changes in blood pressure, heart rate, levels of biomarkers (NT-proBNP, hsTnT, vWF) before and 6 weeks after the ECV and comparison of cognitive function between patients with persistent AF and control subjects, and within patients with persistent AF, before and 6 weeks after the ECV.

### 2.4. Medical History and Clinical Parameters

Different patient-related data were collected during the initial visit, including age, sex, body height, body weight, education level, smoking status (never, former, current), medications, and comorbidities (history of hypertension, diabetes mellitus, hyperlipidemia, coronary artery disease, valvular disease, peripheral artery disease, and heart failure). Further specific information about AF were assessed, including when and how AF was initially discovered, length of ongoing AF episode, symptom severity according to the European Heart Rhythm Association (EHRA) scale, history of previous cardioversions or catheter ablation procedures. Body mass index (BMI) was calculated from the measured height and weight. Stroke and bleeding risk were assessed using CHA_2_DS_2_-VASc (congestive heart failure, hypertension, age, diabetes mellitus, previous stroke, vascular disease, sex category) and HAS-BLED (hypertension, abnormal renal/liver function, stroke, bleeding history or predisposition, labile international normalised ratio, elderly (>65 years), and drugs/alcohol concomitantly) score, respectively.

### 2.5. Cardiologic Assessment and Surveillance

All AF patients underwent echocardiographic exam within 1 month prior to MRI and received anticoagulation therapy for at least 3 weeks prior to ECV, according to the recommendations [12]. Ambulatory 24 h ECG monitoring was performed in all AF patients within 7 days before the MRI examination to determine the average ventricular rate, to register the total time of an extreme ventricular rate (>110 or <50 beats per minute), and to guide the rate control medications.

The ECV procedure was scheduled usually within 1 h after an initial MRI. Patients were instructed to fast for at least 6 h and avoid smoking before the procedures. Additionally, all patients underwent a 12-channel ECG recording to confirm the AF rhythm. Their arterial pressure was measured using a sphygmomanometer, twice in a lying and sitting position, and the mean value of all measurements was taken as a reference. Mean arterial pressure was then calculated as a sum of 2/3 of diastolic blood pressure and 1/3 of systolic blood pressure. They also underwent a blood test, including lipid profile, N-terminal pro-brain natriuretic peptide (NTproBNP), and von Willenbrand factor (vWF). ECV was performed according to the standard procedure.

Patients were instructed to urgently report if they feel or suspect the return of arrhythmia. Otherwise, they underwent a study follow-up visit the day after ECV and thereafter at regular 7-day intervals. The ECG was recorded prior to each follow-up visit by the general practitioner and was reviewed by one of the researchers (J. K.). We used this type of monitoring to timely and accurately determine the AF recurrence to the best of our knowledge. All patients were scheduled for a second MRI approximately 6 weeks after the first one in order to allow sufficient recovery of left atrial systolic function, as previously published [13].

### 2.6. Cognitive Assessment

Cognitive assessment was performed prior and six weeks after ECV using the self-reported Patient-Reported Outcomes Measurement Information System (PROMIS) Cognitive Function index, which consist of an 8-item short-form [14].

### 2.7. MRI Examinations

All MRI examinations were performed using a 1.5 Tesla scanner (Aera, Siemens, Erlangen, Germany) and the acquisition coil was a 16-channel coil. The patients were placed in the supine position and the head was placed safely in a head coil for fixation to avoid motion artifacts.

The standardised protocol included: 3D flash T1, 3D fluid-attenuation inversion recovery (FLAIR), coronal T2-weighted image, diffusion-weighted image (DWI), susceptibility-weighted image (SWI), followed by 2D pseudocontinuous arterial spin labelling (pCASL) examination. DWI (b: 1000 s/mm^2^) was performed using the following parameters: repetition time, 4000 ms; echo time (TE), 86 ms; and slice thickness, 5 mm. Automatically generated apparent diffusion coefficient (ADC) maps were subsequently reviewed. The sequence parameters for pCASL were as follows: TR, 4600 ms; TE, 20.44 ms; inversion time, 1990 ms; and slice thickness, 4 mm. Intravenous contrast material was avoided. The average brain blood flow values were measured in the 12 areas of interest bilaterally from supratentorial and infratentorial regions [15].

The regions of interest (ROI) included the bilateral basal ganglia, thalamus, frontal and occipital white matter, middle cerebellar peduncle, and cerebellar hemisphere. The uppermost image slice showing the centrum semiovale was used as the landmark, and ROI was placed in the frontal white matter. The ROI was placed in the lentiform nucleus and in the thalamus, bilaterally, avoiding the third ventricle. ROI was circle measuring 1 cm^2^. The voxel size was 1.9 × 1.9 × 4 mm. All patients had the same ROI volume and shape. We used the mirror copy ROI method and anatomical landmarks for determination to provide the consistency for measuring each brain area in both time-points.

MRI examinations were evaluated by two board-certified neuroradiologists (SLK and MMG) with consensus agreement. The observers were blinded to the clinical and laboratory finding of all patients.

### 2.8. Image Analysis

The pre- and post-procedural ASL images in study patients as well as ASL images in controls were analysed with Syngovia software (syngo.via Client 8.6). We measured signal intensity in 12 different brain regions for each subject in each time-point using color maps. The obtained values were expressed as mean ± SD. We also evaluated acute ischemic lesions using DWI and ADC maps.

### 2.9. Statistical Analysis

All numerical variables are expressed as mean ± standard deviation. Frequencies data are presented as absolute numbers and percentages of total in parentheses. Global brain blood flow was calculated as the average measured blood flows in each individual bilateral brain region. Numerical variables were compared using Student’s two-tailed *t*-test for independent (AF patients vs. controls) and dependent samples (changes before vs. after the ECV). Frequency variables were analysed using Fisher’s exact test. A *p*-value of 0.05 was set as the statistical significance threshold. To determine the required sample size, we used already published data [9,10]. Data from the literature suggest a clinically significant change in the primary outcome, i.e., brain perfusion by ≥5% of the population average. To test for such a difference, with a statistical significance level of 0.05 and a study power of 80% (beta 0.2), 10 subjects per group were required. All statistical analyses were performed using Prism 9 for macOS, Version 9.3.1 (350), GraphPad Software, LLC, San Diego, CA, USA.

## 3. Results

We included a total of 41 patients in the study. The patients were divided in two groups, the AF group and the control group, respectively. Two subjects in the AF group (one with successful and one with unsuccessful ECV) left the study before the second MRI scan. Table 1 shows baseline characteristics of the two groups. AF patients and the control group were well matched, except that AF patients had a higher resting heart rate (average 90 vs. 68 bpm, *p* < 0.001) and increased values of NT-proBNP and vWF (mean of 1069 vs. 108 pg/mL, *p* < 0.001, and mean of 1.8 vs. 1.4, *p* < 0.05, respectively). A total of four patients in the control group were taking acetylsalicylic acid for primary prevention prescribed by a family physician, and one patient was on anticoagulant therapy after lower leg deep vein thrombosis. No patient was diagnosed with coronary artery disease, lower limb arterial disease, or obstructive sleep apnea. The current episode of AF was the first diagnosis of this arrhythmia in 80% of patients, with a mean duration of 72 days (20–146). Most patients were symptomatic (EHRA class > 1 in 80%) and anticoagulated with direct oral anticoagulants (88%). According to the ambulatory ECG monitoring, the average ventricular rate of AF patients was well regulated (89 beats per minute) (Supplementary Table S1).

### 3.1. Outcomes of ECV

Cardioversion was successful in 22 out of 25 patients (88%). In six (27%) patients, AF recurrence occurred before the second MRI scan with the median of 10 days (ranging from 1 to 32 days since ECV). Therefore, a total of nine (36%) patients had unsuccessful rhythm control strategy (whether ECV failed or arrhythmia recurred) and were considered and analysed as the ’arrhythmia recurrence’ group.

AF patients without arrhythmia recurrence have exhibited a decrease in resting heart rate after the restoration of sinus rhythm (average 91 vs. 65 bpm, *p* < 0.001). Additionally, a decrease in the value of NT-proBNP (average 979 vs. 284 pg/mL, *p* < 0.001) and an increase in systolic blood pressure (average 136 vs. 146 mmHg, *p* < 0.05) were observed (Table 2). On the contrary, the ‘arrhythmia recurrence’ group exhibited further deterioration in NT-proBNP levels after 6 weeks (*p* = 0.02). Other secondary outcomes in this group were without significant change. There were no periprocedural adverse events.

### 3.2. Brain MRI and ASL

During the follow-up period after ECV, there were no acute neurological symptoms nor new silent brain embolic lesions in any patient.

**Figure 1:**
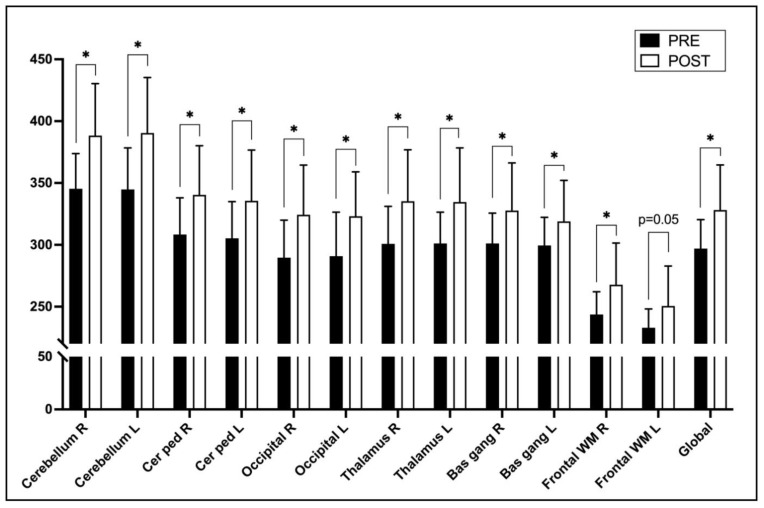
Changes in brain perfusion in different brain regions before (PRE) and 6 weeks after (POST) the electrical cardioversion of atrial fibrillation in patients without arrhythmia recurrence during the follow-up. The measurements were obtained by brain MRI using 2D analysis method [15]. Asterisks (*) denote statistically significant changes (*p* < 0.05; PRE vs. POST; paired *T*-test). Abbreviations: Bas gang—basal ganglia; Cer ped—cerebellar peduncle; Front WM—frontal white matter; L—left side; ns—not statistically significant; R—right side.

Brain perfusion measured by ASL and expressed by signal intensity was significantly higher after ECV in the group without arrhythmia recurrence in all selected brain regions except in the left frontal white matter region where it was at the border of statistical significance (*p* = 0.05). The greatest improvement in perfusion was observed in cerebellar regions (Table 3, Figure 1).

We did not find significant regional differences (*p* = 0.12) in the change in brain perfusion depending on whether the region has been supplied by the internal carotid or the basilar artery. However, there was a tendency for the regions supplied by the posterior vasculature (basilar artery) to have a slightly higher increase in perfusion after ECV than those supplied by the anterior (internal carotid artery)—11.8 ± 13.6% vs. 8.5 ± 13.1%, respectively. Simultaneously, in the group with arrhythmia recurrence, there was no significant change in brain perfusion from baseline in any of the assessed brain regions (*p* > 0.05) (Table 3).

Both study and control groups were similar in morphological characteristics of the brain, except more microbleeding events were observed in the control group (n = 3, 18.8% vs. n = 0, 0.0%, *p* = 0.025).

**Figure 2:**
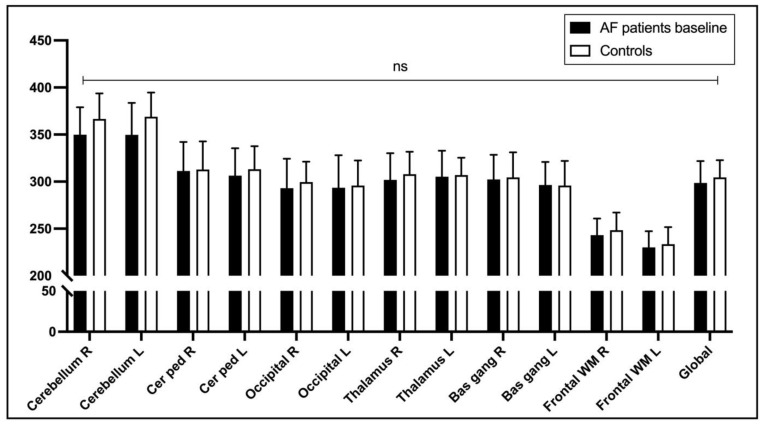
Brain perfusion in different brain regions at baseline in patients with AF and matched control subjects. The measurements were obtained by brain MRI using a 2D analysis method [15]. Abbreviations: Bas gang—basal ganglia; Cer ped—cerebellar peduncle; Front WM—frontal white matter; L—left side; ns—not statistically significant; R—right side.

There was no statistically significant difference in ASL-measured brain perfusion in different brain regions at baseline in patients with AF and matched control subjects (*p* > 0.05) (Figure 2).

### 3.3. Cognitive Function Assessment

Self-reported cognitive assessment did not show any difference between the AF patients and control group in the PROMIS index (52.2 ± 9.6 vs. 51.2 ± 6.2, *p* = 0.71). Additionally, there was no change in cognitive function in the AF group after ECV (53 ± 10 vs. 54 ± 9, *p* = 0.46) (Supplementary Table S2 and Table 2).

## 4. Discussion

This study reports data on the association of persistent AF and brain perfusion parameters compared to matched control subjects, and with regard to subsequent electrical cardioversion. There are several important findings. First, there was no difference in baseline brain perfusion parameters between AF patients and control subjects. Second, the restoration and maintenance of the sinus rhythm was associated with an improvement of MRI brain perfusion parameters in all analyzed regions. This association was further confirmed by observing lack of brain perfusion improvement in patients with unsuccessful sinus rhythm restoration. Finally, there was no association of ECV and cognitive function improvement in patients with AF. Importantly, the study cohort included outpatients with persistent AF that were well characterised in terms of arrhythmia duration, symptoms, ventricular rate control, and adequacy of anticoagulation.

The present study sought to investigate the isolated hemodynamic effect of AF on brain perfusion by comparing AF patients with a control group of patients in stable sinus rhythm and matched cardiovascular risk. While most brain perfusion studies have focused on global and gray matter perfusion, in this study, brain regional perfusion was measured. There are published reports suggesting that certain brain regions involved in cognitive performance, such as the temporal region hippocampus, may be more affected in patients with AF [16,17]. A recent study on elderly patients with AF showed a reduced estimated cerebral perfusion in AF patients compared to subjects in sinus rhythm [9]. That study included participants who on average were ten years older and could have atherosclerotic involvement, such as stroke, which affects cerebral perfusion. Additionally, that study also included patients with permanent AF. Therefore, there are several important distinctions with the present study. Ongoing AF was the first clinical episode of arrhythmia in more than two thirds of patients from this study. One can assume that the autoregulatory mechanisms of cerebral flow maintenance were still preserved in patients with a shorter duration of AF, or the influence of associated comorbidities was more pronounced in the control group. This especially applies to arterial hypertension since usage of antihypertensive medications was shown to be associated with a decrease in cerebral flow [18]. Furthermore, in the control group, we observed a higher number of microbleeding events, a condition associated with long-standing hypertension. Additionally, the incidence of active smokers was higher in the AF group, which may have influenced the higher cerebral perfusion values compared to ex-smokers, as implied by data from one study [19].

As mentioned earlier, cerebral hypoperfusion could be one of the pathophysiological mechanisms through which AF is associated with the occurrence of cognitive impairment. It is well known that AF and cognitive impairment share common risk factors, such as aging, smoking, hypertension, diabetes mellitus, sleep apnea, physical inactivity, vascular disease, inflammation, and heart failure [20]. A large population-based study found that lower cerebral perfusion was related to cognitive impairment, and most cardiovascular risk factors individually influenced that parameter. Except advanced age, use of antihypertensive medications, a high cholesterol level, and active smoking were the most significant confounders for the reduction in cerebral blood flow [11]. It appears that cerebral blood flow may be associated with vascular risk factors as early as midlife [21]. This study implied the importance of other cardiovascular factors in brain perfusion, as well. It was shown that identification and adequate treatment of comorbidities in AF can reduce dementia [22]. Larger longitudinal studies should address the question of how AF differs from other cardiovascular risk factors in terms of its impact on brain perfusion.

The association of AF management and brain perfusion has been previously investigated. Rare studies have evaluated brain perfusion with regard to the focused management of AF involving the restoration of sinus rhythm [10,23,24,25,26] or ventricular rate control [27]. However, the heterogeneity of the population, which included different types of AF and indirect methods for cerebral perfusion measurement, prevented us from drawing strong conclusions. A study by Gardarsdottir et al. [10] showed that the restoration of sinus rhythm by ECV was associated with an improvement in global brain perfusion, whereas the same was not observed in those who remained in AF. However, patients who experienced recurrence of AF after successful ECV were not considered. A recent study by Takahashi et al. [23] reported that patients with non-paroxysmal AF have the greatest benefit in improvement of cerebral perfusion by maintaining sinus rhythm after performing catheter ablation. Patients with recurrences had a further reduction in cerebral perfusion on a control MRI after 6 months. However, this study did not specify the duration of AF, nor the effect of catheter ablation on other outcomes, such as heart rate. In our study, we measured regional brain perfusion by using the same measurement of cerebral microcirculation utilising an ASL method. The restoration of sinus rhythm was associated with an improvement in perfusion in all brain regions, including both gray and white matter regions. By contrast, improvement in cerebral perfusion has not been evident in patients in whom sinus rhythm was not achieved or in which FA recurred. These results highlight the importance of an AF burden since paroxysmal AF was not associated with reduced cerebral blood flow in previous studies [9,23]. Moreover, reduction in brain volume caused by chronic brain hypoperfusion was related to prolonged exposure to arrhythmia [8].

Several studies showed the importance of ventricular rate control in the AF population. In a 10-year follow-up study by Cacciatore et al. [28], low (<50 bpm) and high (>90 bpm) mean ventricular rate responses in patients with AF were indicators of the onset of dementia. A study by Efimova et al. [27] demonstrated that in relatively young patients with difficulty controlling the ventricular rate in AF, ablation of the AV node and implantation of a pacemaker resulted in an improvement in perfusion in some brain regions, although the underlying rhythm remained FA. They speculated that improvement in brain perfusion was due to the increased cardiac output. Although it is not possible to explain the improvement in brain perfusion by increased cardiac output in our study, as all patients had a well-regulated heart rate and preserved left ventricular ejection fraction, we still observed a significant heart rate reduction after cardioversion. One computational study [29] suggested that a higher ventricular response (>90 beats per minute) during AF impairs cerebral haemodynamics by increasing critical cerebral events (hypoperfusion and hypertensive events) at the distal cerebral circle. Cardioversion significantly reduced both hypoperfusive and hyperperfusive/hypertensive microcirculatory events in cerebral circulation [24]. Therefore, the improvement of brain perfusion in sinus rhythm can be attributed to the recovery of the contractile atrial function, improved diastolic filling of the ventricles, and reduced heart rate variability. Results from this study suggest that achieving sinus rhythm results in immediate improvement in brain perfusion, but the lack of difference in baseline brain perfusion between AF patients and control subjects could suggest that, in the long term, other cardiovascular comorbidities have an influence on it, as well. This study did not find a decrease in vWF values after sinus rhythm restoration, which might imply that the endothelial dysfunction is not the main mediator for the cerebral perfusion improvement. A potential clinical implication of these results may suggest a potentially better clinical benefit by the rhythm control approach. In cases of more robust evidence emerges in the future, i.e., by larger longitudinal studies implicating that AF causes chronic hypoperfusion in the brain and thus affects cognitive function, then the most optimal intervention in the prevention of cognitive impairment would be early rhythm control. The importance of the AF burden is highlighted by research indicating that the risk of dementia may be highest in younger AF patients [30]. While in large randomised study, it has been shown that an early rhythm control strategy in AF could have a beneficial effect on cardiovascular outcomes; an impact on cognitive outcomes has not yet been demonstrated [31].

There are several limitations of this study. First, the main drawback is the small number of participants, which could affect the statistical power of the study. In order to avoid the confounding effect of other variables on brain perfusion, one would need a larger group of patients to perform a multivariate analysis. However, we have estimated the sample size according to data from previous studies [9,10]. Second, this was a single center study, which can lead to selection and treatment bias. Third, although the patients were relatively well monitored clinically, it cannot be ruled out that the subjects in sinus rhythm after ECV had short, asymptomatic recurrences of AF. For this purpose, monitoring heart rhythm after ECV using an insertable cardiac monitor would be better. Data [23], however, suggest that paroxysmal episodes of AF are unlikely to affect brain perfusion. Likewise, we could not reliably determine whether patients in the control group had episodes of AF that were not noted in the medical records. We tried to avoid this issue by excluding those who sought medical treatment for any type of arrhythmia. Fourth, an important limitation is that cerebral blood flow was measured indirectly using the signal intensity for quantification of brain perfusion. However, this study aimed to assess variations in brain perfusion regarding ECV rather than determining exact cerebral blood perfusion levels. The main focus was on determining if sinus rhythm restoration improves perfusion in a specific subset of AF patients, and more specifically, what happens to the brain perfusion in the event of recurrent AF.

## 5. Conclusions

This study did not show differences in overall brain perfusion between persistent AF patients and matched control subjects, but restoration and maintenance of sinus rhythm with ECV were associated with significantly improved brain perfusion parameters. There was no association of ECV and changes in cognitive function amongst AF patients. Further prospective studies should investigate the underlying mechanisms for these findings and assess the importance of rhythm control in patients with other AF types.

## 6. Patents

This section is not mandatory but may be added if there are patents resulting from the work reported in this manuscript.

## Figures and Tables

**Table 1:** Subject characteristics.

	AF Patients (N = 25)	Controls (N = 16)	*p* Value
Sex (male)	16 (64%)	10 (63%)	0.99
Age (years)	67 ± 8	66 ± 4	0.77
Height (cm)	178 ± 10	178 ± 11	0.98
Weight (kg)	93 ± 13	90 ± 12	0.59
BMI (kg/m^2^)	29 ± 3	28 ± 3	0.49
SBP (mm Hg)	134 ± 16	136 ± 18	0.77
DBP (mm Hg)	80 ± 8	80 ± 11	0.93
Heart rate (bpm)	90 ± 16	70 ± 11	<0.0001 *
**Diagnostic tests**			
Hgb (g/L)	142 ± 15	146 ± 9	0.34
Htc	0.423 ± 0.043	0.427 ± 0.023	0.79
Creatinine (mol/L)	85 ± 19	79 ± 9	0.27
eGFR (ml/min)	74 ± 20	70 ± 10	0.37
Blood glucose (mmol/L)	6.3 ± 2.5	6.7 ± 2.5	0.67
Albumin (g/L)	43.8 ± 3.3	43.8 ± 2.8	0.96
Total cholesterol (mmol/L)	5.0 ± 1.0	5.4 ± 1.0	0.23
LDL (mmol/L)	3.0 ± 0.9	3.1 ± 0.7	0.59
NT-proBNP (pg/mL)	1069 ± 605	108 ± 72	<0.0001 *
hs-TnT (ng/L)	9.8 ± 4.1	12.4 ± 4.8	0.07
vWF	1.8 ± 0.7	1.4 ± 0.4	0.04 *
PROMIS	52.2 ± 9.6	51.2 ± 6.2	0.71
**Comorbidities**			
Smoking	7 (28%)	4 (25%)	0.99
Hypertension	18 (72%)	12 (75%)	0.99
Diabetes	4 (16%)	3 (19%)	0.99
CKD	0 (0%)	0 (0%)	NS
**Medications**			
Anticoagulation	25 (100%)	1 (6%)	<0.0001 *
Antiplatelets	0 (0%)	4 (25%)	0.02 *
β blocker	23 (92%)	3 (19%)	<0.0001 *
ACE inhibitor	15 (60%)	11 (69%)	0.74
Statin	7 (28%)	4 (25%)	0.99

**Comparison of various subject characteristics measured at baseline between interventional and control group.** Values are expressed as averages ± standard deviation or absolute number and percentage of total (in parenthesis). Differences for numerical variables are analysed using Student’s *t*-test for independent samples, and frequency variables are compared using Fisher’s exact test. An asterisk (*) denotes statistical significance between groups (*p* < 0.05). Abbreviations: ACE—angiotensin-converting enzyme; BMI—body mass index; CKD—chronic kidney disease; DBP—diastolic blood pressure; eGFR—estimated glomerular filtration rate using CKD-EPI formula; Hgb—haemoglobin concentration; hs-TnT—high-sensitive troponin T concentration; Htc—haematocrit; LDL—low-density lipoprotein concentration; NS—not significant; NT-proBNP—N terminal pro brain natriuretic peptide concentration; PROMIS—cognitive test; SBP—systolic blood pressure; vWF—von Willebrand factor concentration.

**Table 2:** Differences in secondary outcomes after electrical cardioversion in patients without arrhythmia recurrence (N = 15 †).

Parameter	PRE	POST	*p* Value
SBP (mm Hg)	136 ± 19	143 ± 18	0.02 *
DPB (mm Hg)	79 ± 9	80 ± 7	0.67
HR (bpm)	91 ± 15	65 ± 8	0.0002 *
NT-proBNP (pg/mL)	979 ± 379	284 ± 145	<0.0001 *
hs-TnT (ng/L)	9.7 ± 4.1	9.2 ± 4.0	0.31
vWF	1.8 ± 0.8	1.7 ± 0.4	0.45
PROMIS.	53 ± 10	54 ± 9	0.46

**One patient has been lost to follow-up.** Values are expressed as mean ± standard deviation. †—one patient was lost to the follow-up. Asterisks (*) denote statistically significant changes (*p* < 0.05; PRE vs. POST; paired *T*-test). Abbreviations: DBP—diastolic blood pressure; hs TnT—high-sensitive troponin T concentration; NT-proBNP—N-terminal pro brain natriuretic peptide concentration; PROMIS—cognitive test; SBP—systolic blood pressure; vWF—von Willebrand factor concentration.

**Table 3:** Changes in brain perfusion in different brain regions before (PRE) and 6 weeks after (POST) the electrical cardioversion of atrial fibrillation obtained by 2D ASL brain MRI imaging in patients with and without arrhythmia recurrence.

	Brain Perfusion					
	without Arrhythmia Recurrence (N = 15)			with Arrhythmia Recurrence (N = 8)		
	PRE	POST	*p* Value	PRE	POST	*p* Value
Cerebellum R	345 ± 26	388 ± 42	0.003 *	352 ±27	369 ± 27	0.23
Cerebellum L	345 ± 35	390 ± 45	0.002 *	351 ± 30	364 ± 26	0.29
Cer ped R	309 ± 31	340 ± 40	0.01 *	307 ± 19	321 ± 30	0.32
Cer ped L	305 ± 31	336 ± 41	0.01 *	301 ± 21	317 ± 33	0.29
Occipital R	289 ± 31	324 ± 40	0.004 *	292 ± 29	302 ± 37	0.48
Occipital L	292 ± 37	323 ±36	0.01 *	290 ± 22	297 ± 36	0.61
Talamus R	303 ± 30	335 ± 42	0.01 *	298 ± 23	304 ± 25	0.70
Talamus L	303 ± 25	335 ± 44	0.01 *	309 ± 32	311 ± 25	0.89
Bas gang R	302 ± 25	328 ± 39	0.03 *	302 ± 32	315 ± 22	0.39
Bas gang L	297 ± 21	319 ± 33	0.04 *	295 ± 27	301 ± 23	0.65
Front WM R	244 ± 19	268 ± 34	0.01 *	240 ± 17	251 ± 19	0.32
Front WM L	234 ± 16	251 ± 32	0.05	230 ± 14	235 ± 24	0.67
Global	297 ± 24	328 ± 37	0.008 *	297 ± 22	307 ± 24	0.45

Two patients (one with and one without arrhythmia recurrence) have been lost to the follow-up. Values are expressed as mean ± standard deviation. Asterisks (*) denote statistically significant changes (*p* < 0.05; PRE vs. POST; paired *T*-test). Abbreviations: Bas gang—basal ganglia; Cer ped—cerebral peduncle; Front WM—frontal white matter; L—left side; R—right side.

## Data Availability

The data used during this study are available on request from the corresponding author.

## Supplementary Materials

The following supporting information can be downloaded at: https://www.mdpi.com/article/10.3390/life13040935/s1, Table S1: Characteristics of atrial fibrillation group, Table S2: Comparison of cognitive function score based on electrical cardioversion, Figure S1: Flow diagram.
