# Supplementary material for: Association of Electrical Cardioversion with Brain Perfusion and Cognitive Function in Patients with Atrial Fibrillation
Source: Life (Basel). 2023 Apr 3;13(4):935. doi: 10.3390/life13040935 (PMC10142430; doi:10.3390/life13040935)

life

MDPI

Supplementary Table S1. Characteristics of atrial fibrillation group.

	**AF Patients (N = 25)**
Duration of AF (days)	72 ± 37
EHRA class	
1	5 (20 %)
2a	11 (44 %)
2b	8 (32 %)
3	1 (4 %)
4	0 (0 %)
24h Holter monitor	
Average HR (bpm)	89 ± 16
Time in tachycardia > 110/min (minutes)	299 ± 309
Time in tachycardia > 110/min (% of time)	22 ± 23
Echo parameters	
LA size (mm)	47 ± 5
LV EF (%)	57 ± 10
CHA_2DS_2-VASc score	2.3 ± 1.1
HAS BLED score	0.7 ± 0.6
Anticoagulation drug	
warfarin	3 (12 %)
apixaban	13 (52 %)
dabigatran	5 (20 %)
edoxaban	3 (12 %)
rivaroxaban	1 (4 %)
Antiarrhythmic therapy	
amiodarone	8 (32 %)
propafenone	1 (4 %)
Previous ECV	7 (28 %)
Previous PVI	1 (4 %)

Descriptive characteristics of patients with atrial fibrillation before electrical cardioversion. Duration of AF represents time elapsed since documented onset of atrial fibrillation until the day electrical cardioversion has been performed. EHRA class is designated according to recent guidelines (12). CHA_2DS_2-VASc score and HAS BLED score are calculated as previously described (12). Values are expressed as averages ± standard deviation or absolute number and percentage of total (in parenthesis). Abbreviations: AF – atrial fibrillation; ECV – electrical cardioversion; LA – left atrium; LV EF – left ventricular ejection fraction; PVI – pulmonary vein isolation.

Supplementary Table S2. Comparison of cognitive function score based on electrical cardioversion.

**Variables**	**Before**	**After**	***p*-Value ***
PROMIS index	52.6±9.6	52.7±9.6	0.952
PROMIS dimension 1: Slower thinking	4.3±0.9	4.2±0.8	0.390
PROMIS dimension 2: Impression of brain thinking impairment	4.2±0.9	4.3±0.8	0.905
PROMIS dimension 3: Need for stronger focus on everyday activities	4.3±0.9	4.4±0.7	0.214
PROMIS dimension 4: Impairment in multi-tasking	4.3±1.0	4.4±0.8	0.439
PROMIS dimension 5: Concentration impairment	4.0±1.1	4.0±1.1	1.000
PROMIS dimension 6: Need for stronger focus to avoid mistakes	4.2±1.0	4.2±1.2	0.335
PROMIS dimension 7: Impairment in idea shaping	4.1±1.2	4.1±1.0	0.689
PROMIS dimension 8: Impairment in number calculation	4.5±0.9	4.5±1.0	0.934

Data are expressed as mean ± standard deviation.

* t-test.

Supplementary Figure S1. Flow diagram.

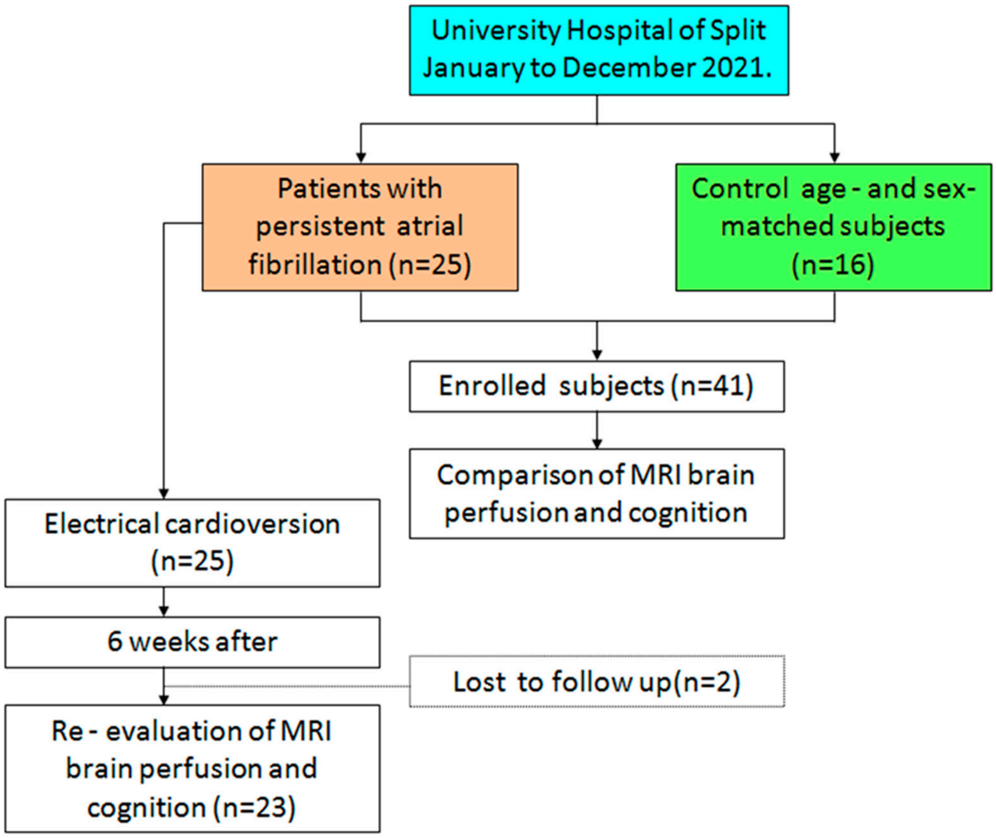

Supplement: Supplementary file 1 [file life-13-00935-s001.zip › life-2260472-supplementary.pdf]
